# Identifying activity level related movement features of children with ASD based on ADOS videos

**DOI:** 10.1038/s41598-023-30628-6

**Published:** 2023-03-01

**Authors:** Xuemei Jin, Huilin Zhu, Wei Cao, Xiaobing Zou, Jiajia Chen

**Affiliations:** 1 South China Academy of Advanced Optoelectronics, South China Normal University (SCNU), Guangzhou, 510006 China; 2 Child Development and Behavior Center, The Third Affiliated Hospital of Sun Yat-Sen University, Guangzhou, 510630 China

**Keywords:** Human behaviour, Computer science, Information technology

## Abstract

Autism spectrum disorder (ASD) is a neurodevelopmental disorder that affects about 2% of children. Due to the shortage of clinicians, there is an urgent demand for a convenient and effective tool based on regular videos to assess the symptom. Computer-aided technologies have become widely used in clinical diagnosis, simplifying the diagnosis process while saving time and standardizing the procedure. In this study, we proposed a computer vision-based motion trajectory detection approach assisted with machine learning techniques, facilitating an objective and effective way to extract participants’ movement features (MFs) to identify and evaluate children’s activity levels that correspond to clinicians’ professional ratings. The designed technique includes two key parts: (1) Extracting MFs of participants’ different body key points in various activities segmented from autism diagnostic observation schedule (ADOS) videos, and (2) Identifying the most relevant MFs through established correlations with existing data sets of participants’ activity level scores evaluated by clinicians. The research investigated two types of MFs, i.e., pixel distance (PD) and instantaneous pixel velocity (IPV), three participants’ body key points, i.e., neck, right wrist, and middle hip, and five activities, including Table-play, Birthday-party, Joint-attention, Balloon-play, and Bubble-play segmented from ADOS videos. Among different combinations, the high correlations with the activity level scores evaluated by the clinicians (greater than 0.6 with p < 0.001) were found in Table-play activity for both the PD-based MFs of all three studied key points and the IPV-based MFs of the right wrist key point. These MFs were identified as the most relevant ones that could be utilized as an auxiliary means for automating the evaluation of activity levels in the ASD assessment.

## Introduction

Autism spectrum disorder (ASD) is a lifelong neurodevelopmental disorder characterized by social communication impairments and restricted, repetitive patterns of behavior^1^. The prevalence of ASD has increased from 6.7 to 23.0 per 1,000 children over the past two decades (from 2000 to 2018)^2^. In addition to the core symptoms, hyperactivity/over-activity is a common clinical joint symptom in children with ASD^1,3,4^, and it seriously affects the quality of life and intervention outcomes for children with ASD^5–8^. Strum et al. reported that 86% of children with ASD had problems regulating activity level^9,10^. Additionally, 41–78% of children with ASD have hyperactivity, impulsivity, and inattention symptoms, similar to attention-deficit/hyperactivity disorder (ADHD)^5,6^. Accurate evaluation of the activity levels in children with ASD is necessary for effective clinical diagnosis, intervention, and daily care.

The activity level evaluation is often included in assessing children screened for ASD, such as in the autism diagnostic observation schedule (ADOS) assessment, a semi-structured and standardized assessment for individuals suspected of having ASD on social interaction, communication, play, and imaginative use of materials^4^. However, the clinicians’ assessment results can be influenced by various factors such as training, resources, and culturally related practices of the clinicians^11^. Taylor et al. conducted a diagnostic reliability study of ADOS videos for 27 professionals. The results showed that only 33% of the video clips received a consistent diagnostic classification^12^. In addition, due to the shortage in the professional clinician workforce, on average, children screened for ASD received their first diagnostic assessment after 48 months and obtained the final diagnosis until 61 months later^13^. With the growing demand for early diagnosis of ASD and the impact of the current COVID-19 pandemic, there is an urgent need for a reliable computer-aided assessment tool based on regular videos to solve the above problems.

In recent years, clinicians implemented information technology (IT) aided methods, including computer vision and machine learning techniques, to assess ASD and accelerate the diagnosis process^14^. Some of these techniques measure children’s attention patterns and communication behaviors by detecting their postures and movements, such as head movement, facial expression^15–18^, hand movement^19,20^, arms flapping, head banging, and spinning^21–23^. Additional studies identified nonverbal social interaction movements by extracting key points^24^ (body skeleton joints) between children and clinicians^25^ or measuring the distance, temporal ratio, and facial orientation^14^. Such studies mainly focused on the core symptoms of ASD, but the related ones on hyperactivity for children with ASD were still minimal.

In an ADOS assessment, clinicians usually evaluate children’s activity level or hyperactivity by looking at whether the children can sit or stand appropriately while participating in predefined activities. We investigated the related research on children’s activity levels and found that the equipment in these studies generally needs to collect information on children’s spatial position, distance, velocity, and micro-movement^26–28^. In addition, some studies for ASD on analyzing infants’ early motor development show that it is very important to detect the motor trajectory of the infant’s limbs and compute their motor features (e.g., standard deviation, mean velocity, and mean acceleration of centroid of motion)^29,30^. Therefore, we believe that it is essential to detect the motion information (e.g., position, distance, micro-movements, and velocity) of children’s bodies and calculate their movement features for activity-level research of children with ASD. However, the existing research often required peripheral devices, such as infrared cameras^26^, Impulse-radio ultra-wideband radars^27^, and accelerometer devices^28^, some of which needed to be worn by the children. This intrusive method may cause children with ASD to be stimulated or feel uncomfortable, affecting the final ratings^31^. In addition, these studies only collected motion information of a single body part. More importantly, they hardly support analyzing the motion features based on the standard clinical assessment videos, like ADOS, for children with ASD.

A wealth of children’s motion parameters can be observed in ASD study using a non-invasive method^24,25,32^. OpenPose is a common and robust tool used in pose estimation of children in ordinary 2D videos compared to many existing approaches^33–37^, e.g., Kinect. In this study, we developed a non-invasive computer vision and machine learning-based framework for analyzing ADOS assessment videos. We extract the key points of the children in the videos with the help of the OpenPose tool^24^ and arrange the key points in chronological order that resembled motor trajectories to quantify movement features (MFs) in different settings (i.e., different body key points and activities). We defined MFs as a series of statistics regarding pixel distance and instantaneous velocity of body parts’ movement extracted from motion trajectories. We aimed to use MFs extracted from motion trajectory information to indicate the activity level of children in ADOS assessment. We segmented the video as multiple activities throughout the ADOS assessment. We calculated Spearman’s correlations coefficients (SCC) between MFs in different settings and activity level scores provided by clinicians and identified the most relevant ones, i.e., both the pixel distance (PD) based MFs of all three studied body parts and the instantaneous pixel velocity (IPV) based MFs of the right wrist key point in Table-play activity, having SCC values greater than 0.6 with *p* < 0.001. These established MFs captured in video recordings that were highly correlated with clinicians’ ratings provided evidence for assessing the activity level of children with ASD and could be used to support future clinical procedures.

## Materials and methods

**Figure 1:**
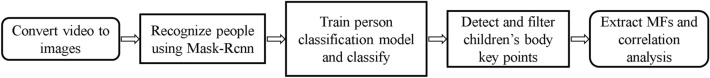
The overview of our research framework.

Figure 1 shows our proposed research framework to identify the most relevant MFs for children with ASD, consisting of 5 consecutive modules, namely (1) converting video into frame images; (2) recognizing people by using Mask-Rcnn^39^, including three sub-steps, namely (a) masking the environment and retaining human, (b) bounding human boundary with rectangular box, and (3) saving boundary box coordinates; (4) training person classification model and classifying person; (5) detecting people’s key points on the categorized images using OpenPose^24^ and filtering the selected key points; (6) extracting participants’ MFs and calculating Spearman correlation coefficient of the MFs with activity level scores. This chapter presents participants’ data collected for the study, data processing, and MFs definition for various modules of the proposed research framework.

### Data description

The video datasets used in this study are ADOS assessment processes^4^ recorded in a clinical context. The videos were segmented according to the game activities, each marked with the start and end times. For each activity, MFs of different body parts were calculated. The details of video datasets are presented below.

### Participants

The analytic samples recorded in ADOS video datasets include 52 participants (42 boys and ten girls aged 2 to 7 years old) with an average age of 3.28 and 1.15 standard deviation (SD). Each video recorded an overall assessment process for one child. Children in the present study were recruited by the Child Development and Behavior Center of the Third Affiliated Hos-pital of Sun Yat-sen University. The study was conducted according to the guidelines of the Declaration of Helsinki, and approved by the Ethics Committee of The Third Affiliated Hos-pital of Sun Yat-Sen University (protocol code 2019[02-247-02]).” Informed consent was obtained from all participants’ parents or legal guardians involved in the study before data collection. Every child’s assessment record had a score reflecting their activity level rated by professional clinicians. The participants’ characters and score definitions are shown in Table 1.

**Table 1:** Participants’ characters and score definition.

Activity level scores	*N*	Male (female)	Age range (years)	Average age in years (*SD*)	ADOS range	Average ADOS total (*SD*)	Score definition
0	18	14 (4)	2.18–7.29	3.27 (1.16)	4–20	15 (4.7)	Be able to sit or stand appropriately when expected to do so in the assessment
1	25	21 (4)	2.14–6.49	3.28 (1.17)	5–22	15 (4.6)	Be able to sit or stand still for a short time when expected, except for the Snack, such as functional and symbolic imitation and birthday party
2	9	7 (2)	2.08–4.28	3.4 (1.2)	16–22	16 (4.1)	Be almost impossible to hold still, except for the Snack

### Correlation power analysis

We conducted a power analysis to confirm if we had a sufficient sample size to achieve the goal of the study. We focused the power analysis on correlational outcomes. Because the study aimed to identify MFs with the strongest correlations with children’s activity levels, we set a minimum threshold of *r* = 0.50 for the power analysis. In addition, to account for multiple comparisons among the MFs, we set the significant level at *p* < 0.0125, assuming four MFs with the strongest correlations (1/4 of a conventional *p* < 0.05 significance level). The power is 0.914, with a minimum correlation of 0.50.

### Video segmentation and activity definition

**Figure 2:**
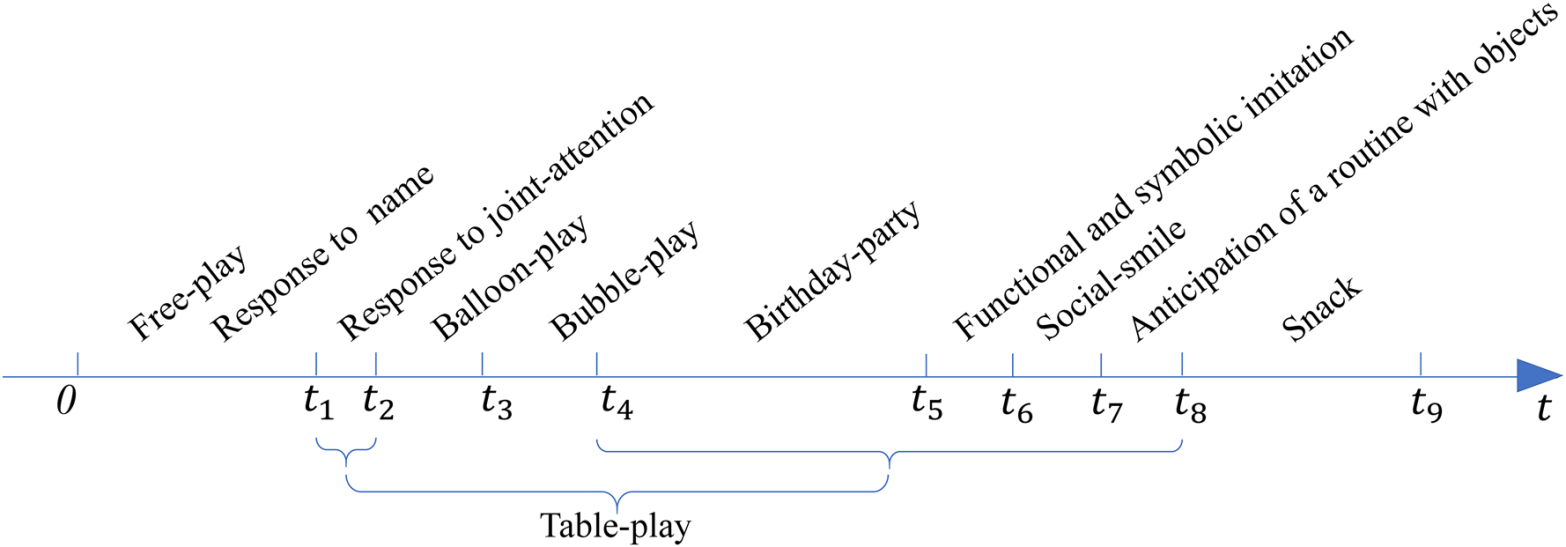
10 activities segmented in the time domain for each video.

The videos were recorded to observe and assess children’s referral to ADOS Module 1. ADOS Module 1 is intended for children over 24 months of age with nonverbal language^4^. Each video included ten activities (see Fig. 2), i.e., Free-play, Response to name, Response to joint attention, Balloon-play, Bubble-play, Birthday-party, Anticipation of a routine with objects, Functional and symbolic imitation, and Snack. The participants usually need about 30–50 min to complete all ten activities. In this study, we segmented the videos by marking each activity’s start and end times. Figure 2 shows how the activities are segmented in each video. In Free-play, it did not need children to sit or stand properly. The Response to name is usually included in the Free-play. The Snack is not for assessing the activity level. The period between ${t}_{1}$ and ${t}_{8}$ including 7 activities, except Balloon-play and Bubble-play; all five other activities require the participants to sit next to a table to complete the tasks. Therefore, we consolidated these five activities as one combined activity and named it the “Table-play” for further study. Table 2 lists the average and SD of the time occupied by five activities in the studies videos: Table-play, Response to joint attention (referred to as Joint attention in later sections), Birthday-party, Balloon-play, and Bubble-play. Each scenario of Table-play involving the assessor sitting next to the table and the toys presented on the table begins with the child returning to the table, where the origin of the coordinate system is, making it more appropriate to assess the child’s activity level throughout the space.

**Table 2:** The time duration for 5 activities (Unit: minutes).

	Table-play	Joint-attention	Birthday-party	Balloon-play	Bubble-paly
Average (*SD)*	11.89 (5.68)	1.08 (0.87)	5.33 (2.22)	2.73 (1.12)	2.96 (1.17)

### Data processing

The participants’ bodies’ motion trajectory information must be prepared for MFs calculation. For processing the video datasets, we first converted the videos into frame images, then located the position of participants in each converted frame image, and finally used the key points extraction tool OpenPose^24^ to obtain the body key points of the participants.

### Video conversion

We first converted the ADOS assessment videos to 10 frames/second frame images and then used Mask-Rcnn^39^ to recognize people on each frame image. In this step, the mask images and bounding box were cut off and saved with the bounding-box coordinates simultaneously. The Mask-Rcnn tool provides the necessary building blocks for easily creating detection and segmentation models using PyTorch 1.0^39^. Its average precision for person segmentation is very well (see Section S6 of the supplementary materials). Here we use its demo model to detect and segment persons on the images.

### Training classification model and predicting

For each video, we selected 60 images by sampling at an equal interval. Specifically, for a video with *N* frames, the interval for extracting the images is *N*/60. Each image is further extracted to several masked samples, each with only one person. We labeled the masked samples into different categories (e.g., participants, clinicians, or parents). The total frame, sample interval, number of masked samples for training set, number of masked samples for validation set, and maximum number of person categories for the selected 20 videos are listed in Table S1 of the supplementary materials. We randomly divided the labeled masked samples into a training and validation set with a ratio of approximately 5:1. In the training process, we implanted ResNet-152 (152-layer Residual Neural Network) on the PyTorch1.0 benchmark to train the person classification model for each video. The flow chart demonstrating the network structure and transmission features is shown in Section S1 of the supplementary materials.

During the training process, we set the total epochs and batch size to 200 and 16, respectively. The best classification model was saved and used to classify person on all mask images. The best classification model is determined according to three training performance metrics one by one: (1) validation accuracy, (2) training accuracy, and (3) validation loss. The models with the highest validation accuracy are first selected. If several models with the highest validation accuracy, the training accuracy is considered the second most important performance metric. If still having multiple models with the highest validation and training accuracy, the best model is determined with the lowest validation loss. One detailed example is included in Section S2 of the supplementary materials.

After training, the classification models were used to identify person on all mask images for each video. Each mask image had a corresponding bounding box image. When the mask images were classified by the models, the classification results of the mask images were also valid for bounding box images. According to the boundary coordinates, the classified bounding box images were pasted onto the black background images in the same position as the original images.

### Key points generation and filter

After the above steps, we got the children’s images with a gray background. OpenPose was used to detect persons’ key points on images just containing the participating children^24,40^. OpenPose is a pose estimator tool that can work on 2D/3D video, image, or webcam. High accuracy fitted this study. Therefore, we chose the BODY_25 model as the default setting and ran the “OpenPoseDemo.exe” with the configuration as “—net_resolution “1312 × 736”—scale_number 4—scale_gap 0.25—hand—hand_scale_number 6—hand_scale_range 0.4”.

Because of overlaps of multiple persons, other persons might appear in the child’s bounding box images. So, multi-person’s key points may be recognized by OpenPose. A simple key point filtering algorithm is used to screen the key points of the child (see Section S3 of the supplementary materials). Finally, we got the trajectory information of the child’s key points.

### Movement feature definition

The clinicians give activity level scores of the participants according to the score definitions shown in Table 1. We used the PD-based and IPV-based MFs to quantify the participants’ motions in ADOS assessment videos. In this section, we describe the motion information and MFs calculation using the motion information.

### Motion information

**Figure 3:**
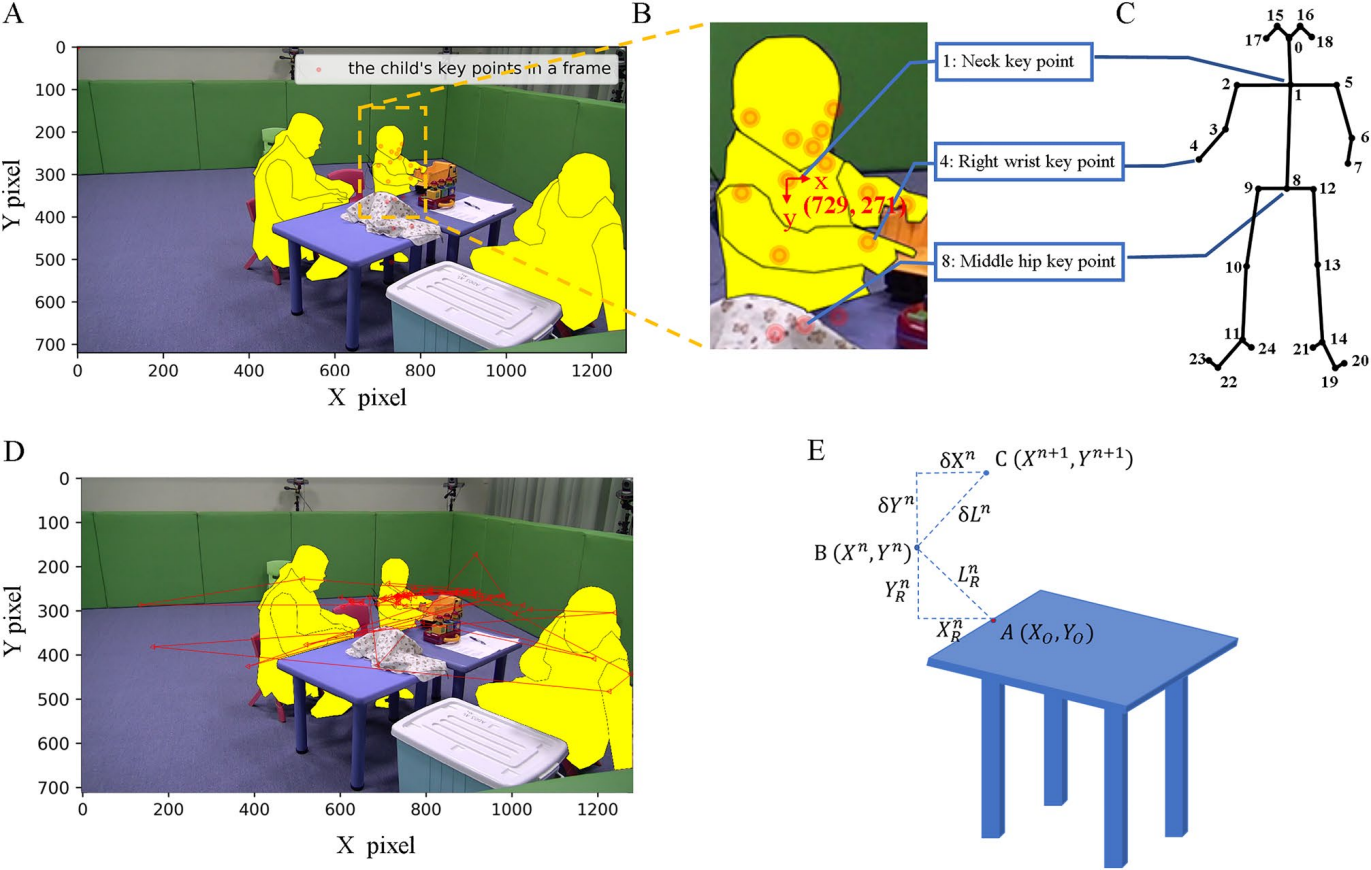
(**A**) A child’s key points in one frame image. (**B**) The local image of the child in Fig. 3A includes his whole-body key points and his neck key point (referring to key point 1) pixel coordinates. (**C**) The corresponding relationship between human joins and 25 key points extracted by OpenPose^24^. (**D**) The trajectory tracking of the neck key point of a child in the view of the camera with an interval of 20 s during ${t}_{1}$ and ${t}_{8}$. (**E**) The pixel distance (PD) and instantaneous pixel velocity (IPV) calculation process for the neck key point.

Here, we define the pixel coordinates of children’s body key points as their motion information. One example of the motion information in our study can refer to the child’s body key points in one frame (Fig. 3A) and the local image (part of the frame that includes the participating child, Fig. 3B). Figure 3C shows the human body joints map to in total 25 key points extracted by OpenPose. We focused on three key points, i.e., neck (key point 1 defined in OpenPose), right wrist (key point 4 defined in OpenPose), and middle hip (key point 8 defined in OpenPose), which can best present the activity level in a sitting situation. These three parts include both ends of the human spine and the commonly used right wrist. The motion trajectory information of a single key point obtained by the framework is shown in Fig. 3D. The motion trajectory information of the three key points is used to calculate the MFs, which include the average and standard deviation of both pixel distance and instantaneous pixel velocity.

### Movement features (MFs)

Figure 3E is taken to illustrate pixel distance (PD) and instantaneous pixel velocity (IPV) MF calculations. In Fig. 3E, point A is the center of the table’s edge next to the participant, defined as the origin point, point B is the neck key point’s coordinates of a child in the frame of the video captured at the sampled time *n*, and point C is the neck key point’s coordinates of a child in the frame captured at the sample time $n+1$. ${X}_{R}^{n}$ and ${Y}_{R}^{n}$ are the PD between point B and point A on the x-axis and y-axis, respectively. ${L}_{R}^{n}$ is the direct PD between point B and point A in frame $n$. ${\mathrm{\delta X}}^{n}$ and $\updelta {Y}^{n}$ are the IPV between point B (in frame $n$) and point C (in frame $n+1$) in x-axis and y-axis, respectively, and $\updelta {L}^{n}$ is the IPV between point B and C. ${L}_{mean}$ and ${L}_{std}$ are the mean and standard deviation of ${L}_{R}^{n}$, which are the MFs based on PD and can be expressed as:

1 $${L}_{R}^{n}=\sqrt{{\left({X}_{R}^{n}\right)}^{2}+{\left({Y}_{R}^{n}\right)}^{2}},$$

2 $${L}_{mean}= \frac{{\sum }_{i=0}^{n}{L}_{R}^{n}}{n},$$

3 $${L}_{std}=\sqrt{\frac{{\sum }_{i=0}^{n}{\left({L}_{R}^{n}-{L}_{mean}\right)}^{2}}{n}.}$$

${\delta L}_{mean}$ and ${\delta L}_{std}$ are the mean and standard deviation of $\updelta {L}^{n}$, which are the MFs based on IPV and can be expressed as:

4 $$\updelta {L}^{n}=\sqrt{{\left({\mathrm{\delta X}}^{n}\right)}^{2}+{\left(\updelta {Y}^{n}\right)}^{2}},$$

5 $${\delta L}_{mean}=\frac{{\sum }_{i=1}^{n}\left|\updelta {L}^{n}\right|}{n-1},$$

6 $${\delta L}_{std}=\sqrt{\frac{{\sum }_{i=1}^{n}{\left(\updelta {L}^{n}-{\delta L}_{mean}\right)}^{2}}{n-1}}.$$

## Results

Using the proposed framework, we detected the motion trajectories information and calculated the MFs of the children. We used Pearson’s correlation coefficient (PCC) to determine the independence between the MFs. To explore the relationship between the MFs and activity level, we used Spearman’s correlation coefficient (SCC) to correlate the MFs with the activity level scores of the samples and identified the most relevant MFs. We did a correlation power analysis to confirm sufficient sample size.

### Movement features characteristics

**Figure 4:**
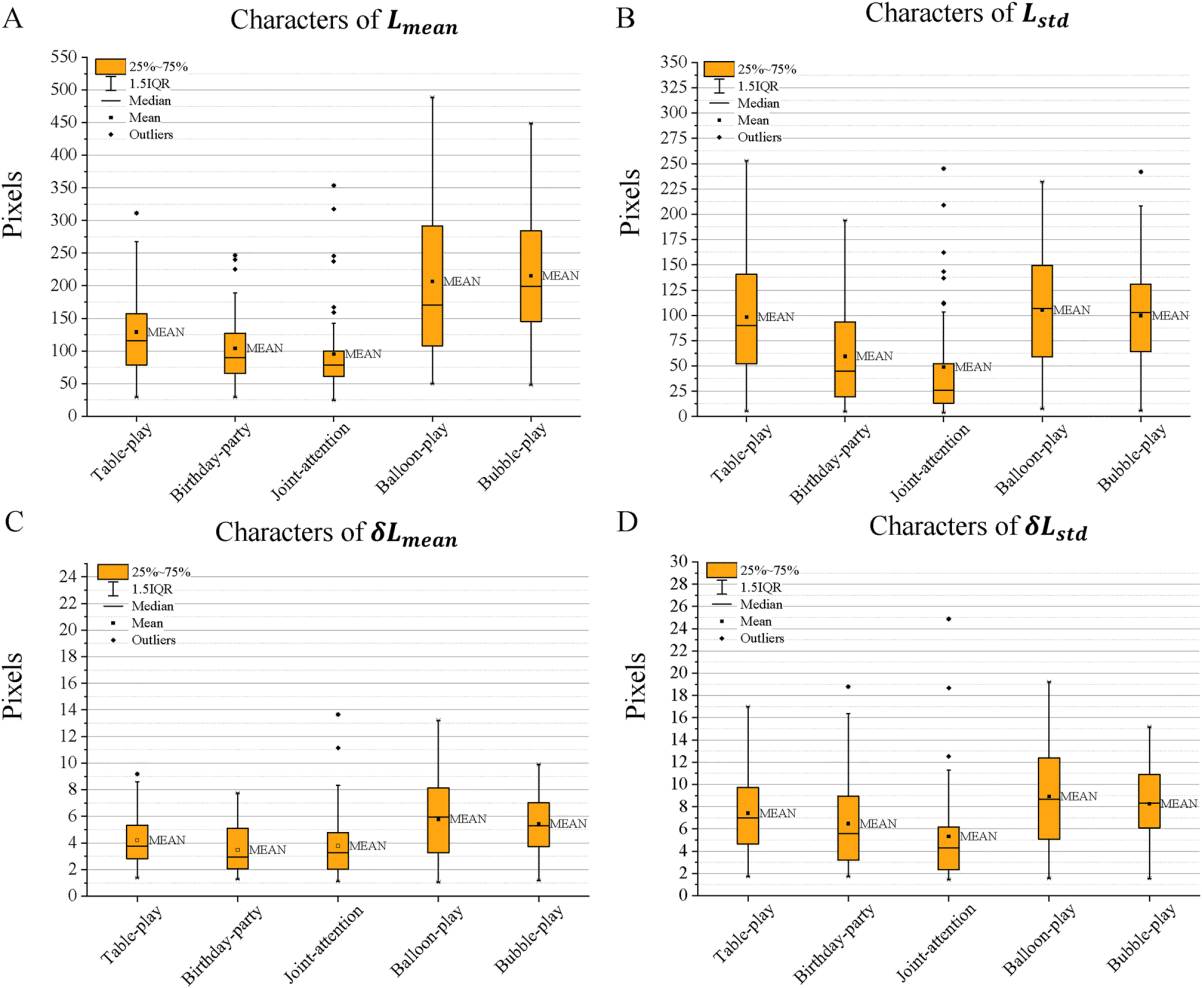
The characteristics of four MFs at the neck key point in different activities: (**A**) ${L}_{mean}$, (**B**) ${L}_{std}$, (**C**) ${\delta L}_{mean}$, and (**D**) ${\delta L}_{std}$.

We calculated four MFs for key points of the child’s neck, right wrist, and middle hip in all considered six items, including the Table-play, Birthday-party, Response to joint attention item, Balloon-play, and Bubble-play. The characteristics of the MFs of different key points in different activities include the range, mean, median, and standard deviation. There are four subplots in Fig. 4 showing the distribution of the MFs of ${L}_{mean}$, ${L}_{std}$, ${\delta L}_{mean}$ and ${\delta L}_{std}$ at the children’s neck key point. The results show that the average and median values of the MFs in Balloon-play and Bubble-play are higher than those in Table-play, Birthday-party, and Joint-attention. It implies that children are more active in these two activities than in the others. The range of 25%-75% is larger in Balloon-play and Bubble-play than in the others, reflecting more variations. We found a similar trend for MFs characteristics at the other key points (see Figs. S4 and Fig. S5 in Supplementary Materials).

### Pearson’s correlation coefficient between movement features

To determine the independence of MFs and eliminate the MFs with high correlation, we conducted the PCC analysis between the MFs. First of all, we carried out the PCC calculations between different MFs, i.e., ${L}_{mean}$, ${L}_{std}$, ${\delta L}_{mean}$ and ${\delta L}_{std}$, in a specific activity for a specific key point (for example, the Table-play activity and the neck key point). The PCC values are in the range of 0.5 and 0.8, showing a strong correlation (see Table S6 in Supplementary Materials). Then, we carried out the PCC between the same MFs but in different activities for the same key point. The PCC results are in the range of 0 to 0.7, showing a medium correlation or weak correlation (see Table S7 in Supplementary Materials). Finally, we performed the PCC calculation between the same MFs in the same activities but for different body key points. The PD-based MFs ${L}_{mean}$ and ${L}_{std}$ of all three considered body key points have PCC values of almost 1, while the IPV-based MFs ${\delta L}_{mean}$ and ${\delta L}_{std}$ of the three body key points have PCC values in the range of 0.6 to 0.9, see Table 3.

**Table 3:** Pearson’s correlation coefficients between the different body parts’ MFs in the Table-play activity.

MFs	Key points	Neck	Right wrist	Middle hip
${L}_{mean}$	Neck	1	0.98	0.95
	Right wrist		1	0.98
	Middle hip			1
${L}_{std}$	Neck	1	0.99	0.99
	Right wrist		1	0.98
	Middle hip			1
${\delta L}_{mean}$	Neck	1	0.92	0.7
	Right wrist		1	0.64
	Middle hip			1
${\delta L}_{std}$	Neck	1	0.78	0.85
	Right wrist		1	0.75
	Middle hip			1

In this study, we have eliminated the MFs with high similarity. For the Table-play activity, the PD-based MFs ${L}_{mean}$ and ${L}_{std}$ of the neck, middle hip, or right-wrist key points are highly correlated, and hence we only retained the PD-based MFs of one body key point. The neck was selected for further research because the neck key point is more visible in ADOS videos compared to the other key points, resulting in higher accuracy. In addition, we retained IPV-based MFs of three key points for the PCC results between the MFs of different key points.

### Relationship between movement features and activity level scores

To explore the relationship between children’s MFs and their activity levels, we used Spearman’s correlation to correlate the MFs with the activity level scores of the samples. We presented the results in the following steps:

1. We correlated the sample’s MFs ${L}_{mean}$, ${L}_{std}$, ${\delta L}_{mean}$ and ${\delta L}_{std}$ with the activity level scores in Table-play, Joint-attention, Birthday-party, Balloon-play, and Bubble-play activities. Meanwhile, we also calculated the significance of the SCC results, corrected the significance with BH (Benjamini-Hochberg, a method for multiple comparison correlation) on each activity, and obtained the final *p*-value.
2. By comparing the SCC results, we selected the most relevant MFs, the activities, and body key points for activity level evaluation.
3. We calculated the probability of recognition errors in the selected videos to verify the reliability of the method.

### Study on different activities

**Figure 5:**
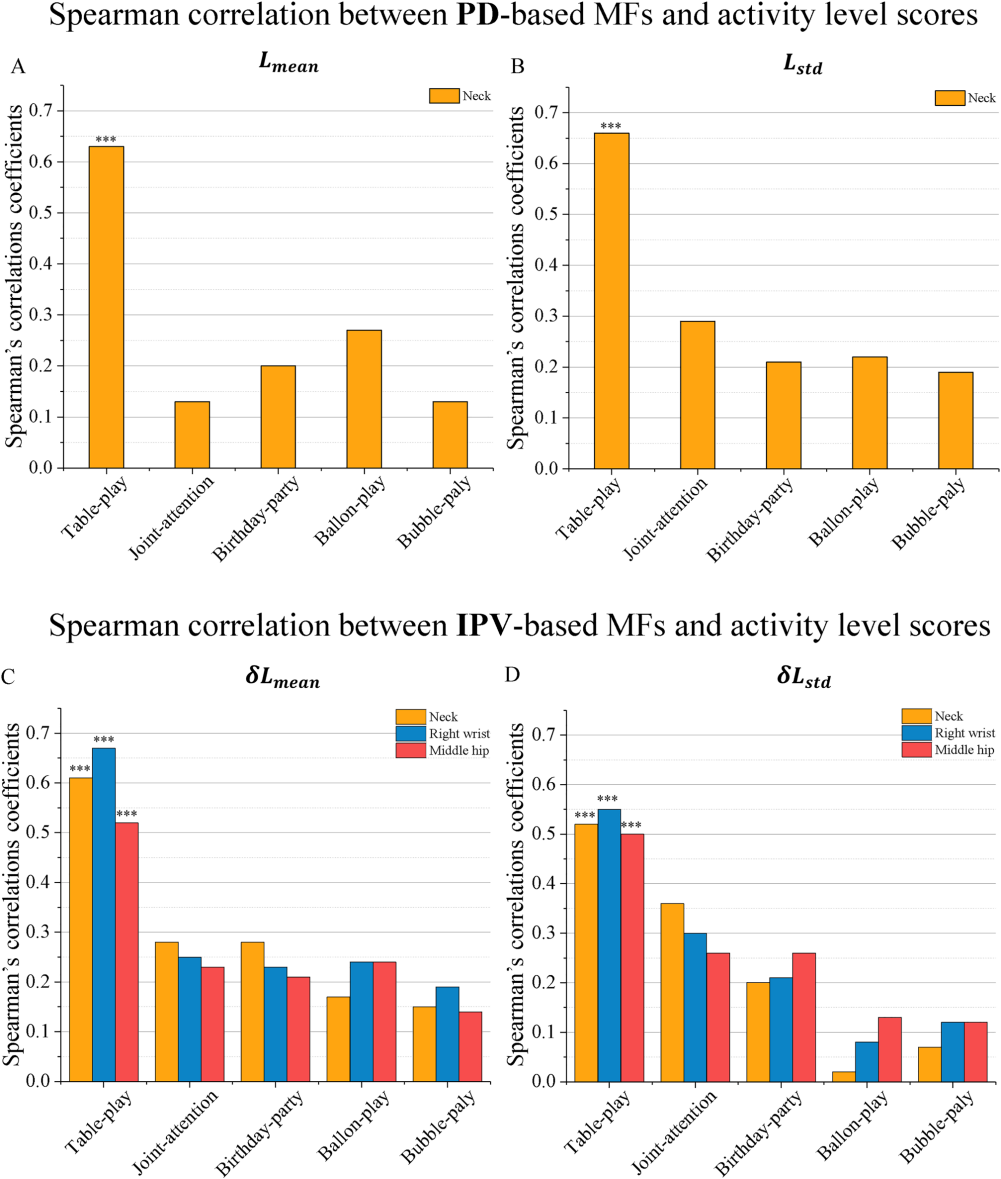
Spearman correlation coefficients between MFs and activity level scores evaluated by the clinicians for all five considered activities. Subfigure (**A**,**B**) showed the Spearman’s correlation coefficient (SCC) between PD-based movement features (${L}_{mean} {\& L}_{std}$) at neck key point and activity level scores; Subfigure (**C**,**D**) showed the SCC between IPV-based (${\delta L}_{mean} \& {\delta L}_{std}$ ) and activity level scores at three body key point (Neck, Right wrist, and Middle hip). *p < .05, **p < .01, ***p < .001.

Figure 5 shows the SCC between MFs and activity level scores for all five selected activities and four MFs, i.e., ${L}_{mean}$, ${L}_{std}$, ${\delta L}_{mean}$ and ${\delta L}_{std}$, where each subplot includes the *p*-value. It can be seen that for various activities, the correlations between the MFs and the activity level are different. The MFs in the Table-play show a strong correlation with the activity level, having an SCC between 0.5 and 0.67. In contrast, the MFs in other activities have a relatively weak correlation with an SCC below 0.5.

Referring to the activity level scoring definition that whether sitting or standing appropriately was the basis for evaluating the activity level, the Table-play includes the activities the participants were expected to interact with the evaluator near the table. Therefore, the MFs in the Table-play activity are more suitable for evaluating children’s activity levels.

### Study on types of movement features

We concentrated on the Table-play activity that demonstrated the greatest correlation with the activity level scores identified in the previous section and studied the impact of various MFs on the SCC results. It should be noted that the four MFs represent different meanings. ${L}_{mean}$ is the average value of PD, ${L}_{std}$ is the standard deviation of PD (representing the dispersion degree of PD), ${\delta L}_{mean}$ is the average value of IPV, and ${\delta L}_{std}$ is the standard deviation of IPV (representing the dispersion degree of IPV). The results reflect that children’s average value and dispersion degree of PD, i.e., ${L}_{mean}$ and ${L}_{std}$, and the average value of IPV, i.e., ${\delta L}_{mean}$ are positively correlated with the activity level, showing a stronger correlation with the activity level scores (greater than 0.6) than that compared with ${\delta L}_{std}$ (below 0.55). The studies suggested that the PD-based MFs and the average value of the IPV are more suitable to reflect the activity levels.

### Study on key points

The MFs of different body key points show different correlations with the activity level scores. For the PD-based features (i.e., ${L}_{mean}$ and ${L}_{std}$), different body parts are highly correlated (see PCC results in Table 3). It is because the PD-based MFs reflect the distance in the pixels between the participant and the original position (i.e., the middle point at the edge of the table near the participant), and these absolute values are large, which makes small changes in different body parts have minor effects on the distance. Therefore, we just calculated the SCC of the neck key point (see Fig. 5A,B). On the other hand, for the IPV-based MFs (i.e., ${\delta L}_{mean}$ and ${\delta L}_{std}$), different body parts introduce obvious variations in the SCC results and hence include three key points, i.e., neck, right wrist, and middle hip, for comparison purposes in Fig. 5C,D. That is because the IPV-based MFs correspond to the movement velocity of the measured body parts. For the same movement, the body parts could change quite differently in the distance and cause the speeds of different body parts to vary a lot. Taking a look at the Table-play activity, it can be found that the SCC between the PD-based MFs and activity level scores for neck key point has strong correlation (> 0.6, see Fig. 5A,B), while the SCC between IPV-based MFs and activity level for the right wrist key point show stronger correlation (0.67 and 0.55, respectively) comparing to that for the neck and middle hip key points (see Fig. 5C,D).

Based on the above results, we considered all three factors, namely the game activities, types of MFs, and key points, and studied how they affect the correlation between the MFs and activity levels. The summary of the SCC results is as follows:

1. The strong positive correlation between MFs (include ${L}_{mean}$, ${L}_{std}$ and ${\delta L}_{mean}$) and activity level scores indicate that the children with higher activity level scores are farther away from the origin position, their activity area is relatively large, and their average instantaneous velocity is faster than those with lower activity level scores.
2. The Table-play activity always shows the strongest correlation among all the considered game activities.
3. From MFs’ and key points’ perspectives, the PD-based ones (including both ${L}_{mean}$ and ${L}_{std}$) do not distinguish among different key points so that the neck key point can well represent the movement features. On the other hand, the IPV-based MFs ${\delta L}_{mean}$ of right wrist key points show the strongest correlation and can be identified as the most relevant one.
4. The most relevant MFs identified in this study have SCC values all greater than 0.5, i.e., the minimum threshold for the power analysis, indicating a sufficient number of samples have been carried out for the research.

### Accuracy of the motion information

The key points of other people in some frames may be mistaken and incorrectly counted as the key points of the participating children, so we evaluated the mismatch rate to understand the data quality of the collected key points. Up to 100 images were randomly selected for each video and labeled whether the child’s key points obtained by OpenPose matched the child’s position in each image. If the key points did not match the child’s joints and belonged to other people, such key points were regarded as a mismatch. We show the mismatch rate for all samples in Table S3 of the supplementary materials. The mismatch rate of all investigated videos ranged from 0 to 13%, with an average value of 2.58% after using the filtering algorithm. The samples with a high mismatch rate are the videos with a third-person (e.g., a parent/guardian) participation who sometimes covered the children. It is also a limitation of our framework, which can affect the quality of the motion trajectory information.

We believe that 2.58% as the average mismatch rate shows relatively high accuracy, particularly for the complex multi-person interactive ADOS assessment videos. We also found that the accuracy could be improved by using the filtering algorithm. Moreover, with less overlap of the participant and their parent(s), the mismatch rate can be close to 0 regardless of using the filtering or not.

## Discussions and conclusions

ASD is a neurodevelopmental disorder that affects about 2% of children, leading a heavy burden to the family. Driven by the shortage of clinicians, a convenient and effective tool for ASD diagnoses is in urgent need. The present study developed a video-based approach leveraging computer vision and machine learning techniques to effectively assess the MFs and objectively estimate the activity level of children with ASD. We chose the videos from ADOS to identify four MFs based on PD and IPV from three parts of the body (neck, right wrist, and middle hip). We analyzed the association between the quantity of MFs and their activity level by calculating the SCC between MFs and activity level scores given by clinicians.

### Summary of results

Our results show that the present framework could quantify human movement from ADOS videos, and the best performance appeared in the PD-based MFs (${L}_{mean}$ and ${L}_{std}$) of all three studied key points and the IPV-based MF (${\delta L}_{mean}$) of the right wrist in the Table-play activity. We discussed the results below and presented our opinions.

### Regarding different activities

Our results demonstrated that the MFs in the Table-play activity strongly correlated with activity level scores. The Table-play activity we defined combined five activities where clinicians require the children to sit near the table to join. Clinicians score children’s activity levels by observing their performance in activities, e.g., whether they can sit or stand properly to participate in the assessment^1,4^, see Table 1. This evaluation method is similar to two hyperactivity-related studies implemented in Refs. 26 and 27, in which children are required to participate in continuous performance testing (CPT). Both assessment activities (CPT and Table-play) require children to be able to participate in a designated location. In comparison, the correlations between MFs and activity level scores were lower in Balloon-play and Bubble-play, in which the children were encouraged to move around. The above discussion suggests that the MFs in our defined Table-play activity are highly associated with clinical judgments and can reflect children’s actual activity levels.

The duration of the game activity may also be a factor in evaluating the correlation. The Joint-attention and Birthday-party are parts of the Table-play and hence obviously shorter than the Table-play. Especially, the Joint-attention activity spends 1.08 min on average (see Table 2), around one-tenth of the Table-play activity. As shown in Fig. 4, there are many outlier points in the Joint-attention activity, which cannot stably reflect the activity level of the participating children. It implies a longer assessment time may result in fewer outliers and easier for converging to a normal distribution, leading to a higher correlation between the MFs and activity level. This finding is similar to the outcome from Li et al.^27^, which shows mean function of the activity level of the ADHD group slightly increased over time with high dispersion when the evaluation time was longer ~ 7 min compared to the healthy control groups. Therefore, we hypothesize that when studying children’s activity levels through the ADOS videos, the duration of the activity should be long enough to get more reliable results.

### Regarding different chosen key points

Our study has found that IPV-based MFs of the right wrist key point achieved the best performance. In our hypothesis, different key points of the body reflected different forms of physical movement. because the wrist motion information includes body and wrist movements, such as body language and tool use. Different from body movements, right wrist movements are more flexible. They could change quite differently in the distance, which can cause the velocity of different body parts to vary dramatically. Yan et al.’s research also supports our findings. They found that the hyperactive children showed more variant movement outcomes in arm movement^41^. Based on extensive research, we believe that the IPV-based MFs of the right wrist can better reflect the children’s activity level compared with the neck and middle hip. In addition, we estimated the correlation between MFs at different key points. PCC results showed PD-based MFs are highly similar and did not distinguish in different studied key points (see Table 3).

### Regarding the movement features

We found that most related research used sensors to collect motion information from the participants in real-time, including distance, speed, and their distribution in time, as positive correlation parameters for the activity level of the participants^26–28^. In our study, the MFs extracted from the ADOS assessment video can also accurately reflect the activity level of participants. The strong correlations between the MFs and activity level scores verify the feasibility of our proposed approach. Compared to many existing studies^26–28^ that request extra sensors for motion tracking, our approach is non-contact and hence friendly to children with ASD.

### Advantages and shortcomings

The above discussion on activity content and duration, key points, and MFs shows that our method is reasonable and feasible. In contrast with the related existing studies on activity level, the performed study has the following advantages: (1) Multiple body parts recognition, which allows simultaneous monitoring and assessing the activity levels of different body parts, (2) Non-invasive, as only using video data sets which include the participants (The way of information acquisition does not affect children’s subjective feelings, which is friendly for children with ASD), and (3) Multiple-person recognition, our computer vision and machine learning-based framework can locate and identify every person in the video and effectively distinguish each person’s motion trajectory information.

Although our framework can effectively quantify the participants’ MFs, which showed a strong correlation with activity levels, there are also some shortcomings. Since the wrist is more likely to be obscured by toys, other figures, or other parts of the child’s body in the video, it also poses a challenge for quantitative estimation of the micro-motion of this key point. In addition, due to the distortion of the camera field of view, the pixel error of micro-motion for the wrist is large when children walk around the room. So, the study on fine wrist movement requires us to introduce multi-cameras data from different angles and synchronize them. Meanwhile, an algorithm must be designed to build a spatial model of wrist motion using multi-angle images. This study focused on the overall movement level of children, and we look at more fine-grained symptom-related behaviors in future studies, such as showing, giving, and other gestures. That may be more important in the fine movement of the wrist, the hand, and the fingers.

## Conclusions

In summary, we demonstrated computer vision and machine learning technologies assisted framework to detect the motion trajectory of the participants’ whole body, extracted MFs from complex multi-person ADOS assessment videos, and explored the correlations between the MFs and activity levels. Specifically, we used Spearman correlations coefficients to quantify the relationship between MFs (including PD-based ${L}_{mean}$ and ${L}_{std}$, and IPV based ${\delta L}_{mean}$ and ${\delta L}_{std}$) and activity level scores in five activities for three body parts (neck, right wrist, and middle hip). From the correlation results, we can see that the Table-play activity showed the best outcome, where the PD-based MFs ${L}_{mean}$ and ${L}_{std}$ of neck key point and IPV-based MF ${\delta L}_{mean}$ of right wrist key point strongly correlate with the activity level scores, having correlation coefficients greater than 0.6 with a *p*-value less than 0.001. Through discussion, we concluded that the MFs under appropriate assessment content and long duration (11 min on average) could well reflect the activity level of children. At the same time, we also discussed the influence of different body parts on the MFs. Our framework is capable of identifying the motion information of multi-body parts of children with ASD in a non-invasive way in the multi-person video. It provides a great potential to perform an objective, rapid and automatic evaluation to effectively alleviate the shortage of clinicians.

## Supplementary Information

Supplementary Information.

## Data Availability

Raw data will be made available upon reasonable request from the authors.
