# Supplementary material for: Identifying activity level related movement features of children with ASD based on ADOS videos
Source: Sci Rep. 2023 Mar 1;13:3471. doi: 10.1038/s41598-023-30628-6 (PMC9975881; doi:10.1038/s41598-023-30628-6)
Supplement: Supplementary file 1 — Supplementary Information. [file 41598_2023_30628_MOESM1_ESM.pdf]

Supplementary Information for "Identifying Activity Level Related Movement Features of Children with ASD based on ADOS Videos"

Xuemei Jin[1], Huilin Zhu[2]*, Wei Cao[1], Xiaobing Zou[2] & Jiajia Chen[1]*
[1] South China Academy of Advanced Optoelectronics, South China Normal University (SCNU), Guangzhou, 510006, China
[2] Child development and behavior center, the Third Affiliated Hospital of Sun Yat-sen University, Guangzhou, 510630, China
* Email: zhuhlin6@mail.sysu.edu.cn; jiajia.chen@coer-scnu.org

S1. Training person classification models.

We implanted Resnet-152 to train the person classification model for each video. The train network is shown in Fig. S1. The model includes Resnet-152 layer and full connection (FC) layer. The Resnet-152 outputs 2048 features into the FC layer. The FC layer includes Linear, ReLU, Linear, ReLU, Dropout, Linear, and LogSoftmax layers.

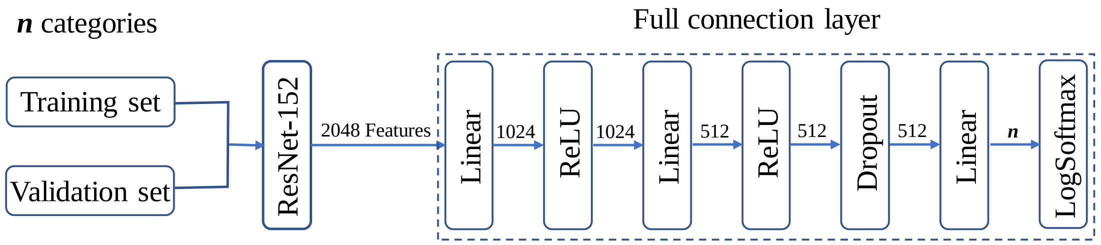

Figure S1. Person classification model network based on ResNet-152. The number on the arrows indicates the number of input features to the next layer.

Table S1 lists key setting parameters for 20 selected videos.

List	Total frame (*N*)	Sample interval (*N*/60)	Number of masked samples for training	Number of masked samples for validation	Maximum number of categories
Video 1	138353	2305	152	30	3
Video 2	150243	2504	142	30	4
Video 3	200627	3343	190	30	4
Video 4	121014	2016	136	30	3
Video 5	82841	1380	150	30	3
Video 6	85884	1431	259	50	4
Video 7	113519	1891	150	50	3
Video 8	96239	1603	150	30	3
Video 9	99525	1658	255	50	4
Video 10	110577	1842	144	30	3
Video 11	89909	1498	219	49	4
Video 12	98935	1648	241	50	4
Video 13	84135	1402	202	40	4
Video 14	88646	1477	145	31	4

Video 15	82154	1369	195	40	4
Video 16	77492	1291	175	40	4
Video 17	60050	1000	153	36	4
Video 18	62756	1045	149	31	4
Video 19	97877	1631	170	38	4
Video 20	67366	1122	116	28	3

Table S1. Key setting parameters for person classification model training , including the total frame number of each video (Total frame N), the sampling image interval (Sample interval N/60), the masked samples of the training set (Number of masked samples for training), the masked samples of the validation set (Number of masked samples for validation set size), the number of classification categories in training and validation sets (Number of categories).

Table S2 lists performance metrics for 20 selected videos.

List	Training accuracy (%)	Validation accuracy (%)	Training Loss	Validation Loss	Number of mask images for Testing	Testing accuracy (%)
Video 1	100	100	0.016659	0.0371	200	99.5
Video 2	100	100	0.0423	0.0373	200	99.5
Video 3	100	100	0.017	0.0351	200	100
Video 4	100	93.33	0.0126	0.2269	200	99.5
Video 5	100	96	0.0178	0.1823	200	99
Video 6	99.61	100	0.0446	0.0418	200	98.5
Video 7	100	100	0.0353	0.0689	200	99
Video 8	99.22	100	0.0826	0.0981	200	97
Video 9	100	98	0.0107	0.0723	200	98.5
Video 10	100	100	0.0215	0.017	200	97.5
Video 11	98.63	95.92	0.0608	0.2095	200	100
Video 12	99.17	100	0.0772	0.0656	200	99.5
Video 13	100	100	0.0398	0.0061	200	99
Video 14	100	93.55	0.0667	0.152	200	100
Video 15	100	100	0.0249	0.014	200	100
Video 16	98.86	100	0.0958	0.1043	200	98.5
Video 17	100	100	0.0399	0.0822	200	100
Video 18	100	93.55	0.0284	0.1393	200	99
Video 19	100	94.74	0.0392	0.1478	200	99.5
Video 20	97.41	100	0.025	0.0963	200	96.5

Table S2. Performance metrics for person classification model training, including training accuracy (Training accuracy), validation accuracy (Validation accuracy), training loss (Training loss), validation loss (Validation loss), the mask image number of the testing set (Number of masked samples for Testing), and testing accuracy (Testing accuracy).

S2. Classification model selection.

The training process of the person classification model for Video 1 is used as an example to illustrate

how to select the optimal classification model. As shown in Fig. S2, when the training accuracy and validation accuracy are optimal (reach 100%), the model with the lowest validation loss is selected as the optimal model. The best model for Video 1 is at Epoch 198, and its training accuracy, validation accuracy, training loss, and validation loss are 100%, 100%, 0.016659, and 0.037091, respectively. The training and validation performance for the selected 20 videos are listed to provide more details (See Table S2). We also include testing results. For the 200 randomly selected masked samples, above 96% testing accuracy can be achieved, and the average testing accuracy is 99.06%.

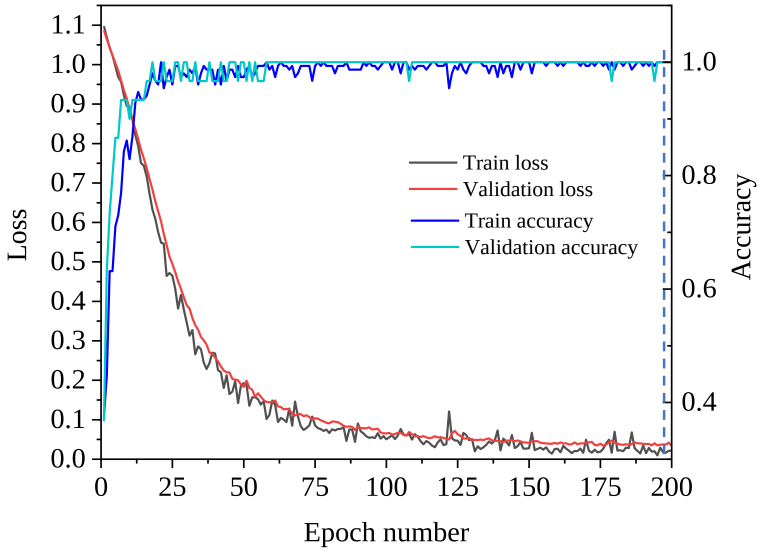

Figure S2. The training and validation performance for Video 1, including training loss, validation loss, training accuracy, and validation accuracy. The vertical dotted line marks epoch 198, which is determined as the best model for Video 1.

S3. Key point filtering algorithm.

The end of the person's limbs in the mask image may be masked, so the bounding box image corresponding to the mask image is used to identify the key points. When cutting the bounding box image, we extend 50 pixels outward on the original border to avoid losing part of the limb image. Because of overlaps of multiple persons, other persons might appear in the child's bounding box images. So, multi-person's key points may be output from OpenPose. We elaborate the filtering algorithm as follows.

1) Mask-Rcnn is used to identify multiple person's mask images and their bounding box coordinate information on an original image. The person classification model is used to label these mask images with categories automatically. Then the mask images' information is stored in an array, including the mask image name, category, classification probability, and the bounding box coordinates (denoted as (x_1, y_1) and (x_2, y_2)).

2) If a mask image is classified as the child, its corresponding bounding box image will be pasted to the black background image for key points recognition in the next step. As shown in Figure S3A of the supplementary materials, two mask images are identified as the child, and their bounding box image, i.e., Bounding box 1 with a classification probability of 0.97 and Bounding box 2 with a classification probability of 0.74, are pasted to black background image. Bounding box 1 is selected as the child's bounding box because of its higher classification probability.

3) All person's key points are extracted by OpenPose. In Figure S3A, two persons' key points

(the parent and the child) are displayed. The total non-zero key point number for Person i in the masked image is denoted as K_i, and the key points number in the bounding box for Person i is denoted k_i. We define the proportion of key points as k_i/K_i. As shown in Figure S3A, the total number of non-zero key points for Person 1 K_1 is 20, and the number of key points in the child's bounding box (i.e., Bounding box 1) k_1 is 19. The proportion k_1/K_1 is 0.95 (19/20). Similarly, the total number of non-zero key points for Person 2 K_2 is 24, and the number of key points in the child's bounding box k_2 is 9. The proportion k_2/K_2 is 0.375 (9/24). By comparing the two persons' proportions, the one's key points with a higher value belong to the child's key points, as shown in Figure S3B.

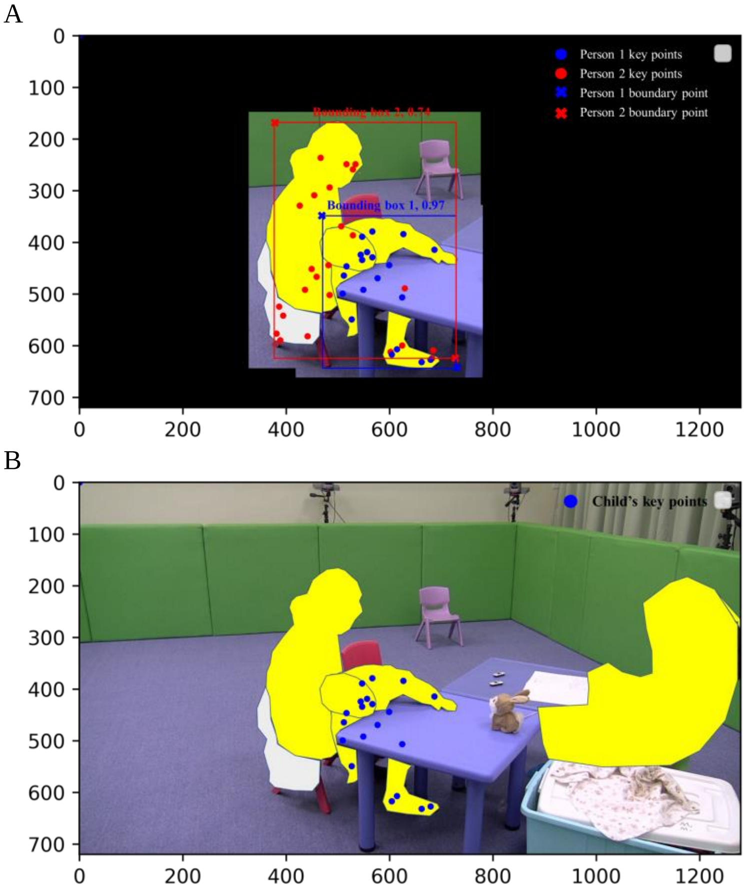

Figure S3. (A) Two persons' key points in an image before filtering. The blue points are the key points of Person 1 (the child), and the blue rectangle is the bounding box of Person 1. The red points are the key points of Person 2 (the parent), and the red rectangle is the bounding box of Person 2. The probability of Bounding box 1 being classified as a child is 0.97, and the probability of Bounding box 2 being classified as a child is 0.74. (B) The child's key point after filtering.

S4. Mismatch of children's key points.

Limited by time and human resources, up to 100 images are randomly selected for each video for accuracy estimate. For some videos, random selection may cause duplication, then less than 100 images were chosen for evaluation. The statistical data includes the image number, the number of mismatched images, the mismatch rate before and after using the filtering algorithm, and the improvement of the filtering algorithm.

List	Image Number	Before Filter		After Filter		Improvement by filtering (%)
		Mismatch Number	Mismatch Rate (%)	Mismatch Number	Mismatch Rate (%)	
Video 1	100	0	0	0	0	0
Video 2	100	5	5	1	1	4
Video 3	99	8	8.08	1	1.01	7.07
Video 4	100	8	8	2	2	6
Video 5	99	2	2.02	1	1.01	1.01
Video 6	100	22	22	10	10	12
Video 7	100	5	5	3	3	2
Video 8	99	5	5.05	4	4.04	1.01
Video 9	99	5	5.05	2	2.02	3.03
Video 10	99	2	2.02	0	0	2.02
Video 11	100	7	7	4	4	3
Video 12	98	1	1.02	0	0	1.02
Video 13	99	1	1.01	0	0	1.01
Video 14	99	1	1.01	1	1.01	0
Video 15	99	1	1.01	0	0	1.01
Video 16	98	4	4.08	1	1.02	3.06
Video 17	97	3	3.09	3	3.09	0
Video 18	100	17	17	5	5	12
Video 19	99	11	11.11	7	7.07	4.04
Video 20	99	2	2.02	2	2.02	0
Video 21	100	1	1	0	0	1
Video 22	99	7	7.07	1	1.01	6.06
Video 23	98	2	2.04	0	0	2.04
Video 24	98	21	21.43	11	11.22	10.21
Video 25	100	1	1	1	1	0
Video 26	100	0	0	0	0	0
Video 27	98	7	7.14	3	3.06	4.08
Video 28	100	6	6	2	2	4
Video 29	98	34	34.69	10	10.2	24.49
Video 30	100	2	2	1	1	1
Video 31	99	14	14.14	5	5.05	9.09
Video 32	100	4	4	1	1	3
Video 33	99	5	5.05	3	3.03	2.02
Video 34	100	11	11	1	1	10
Video 35	100	12	12	3	3	9
Video 36	100	1	1	1	1	0
Video 37	100	5	5	1	1	4
Video 38	100	0	0	1	1	-1

Video 39	100	5	5	2	2	3
Video 40	99	7	7.07	4	4.04	3.03
Video 41	97	6	6.19	2	2.06	4.13
Video 42	100	4	4	2	2	2
Video 43	100	9	9	3	3	6
Video 44	100	8	8	6	6	2
Video 45	98	6	6.12	0	0	6.12
Video 46	99	3	3.03	3	3.03	0
Video 47	99	14	14.14	13	13.13	1.01
Video 48	99	0	0	0	0	0
Video 49	100	5	5	2	2	3
Video 50	99	19	19.19	3	3.03	16.16
Video 51	100	1	1	0	0	1
Video 52	100	3	3	1	1	2
Average			5.5285		2.58	2.95

Table S3. Key points mismatch for all videos.

S5. The comparison of pose estimation software

We compared different body parts' average precision (AP) for the software in Table S4 on the MPII Multi-Person Dataset. In Table S4 we can see that OpenPose is one of the best pose estimation software, and image processing speed is also optimal.

Method	Head	Shoulder	Elbow	Wrist	Hip	Knee	Ankle	mAP	s/image
Full testing set									
DeeperCut	78.4	72.5	60.2	51	57.2	52	45.4	59.5	485
Iqbal et al.	58.4	53.9	44.5	35	42.2	36.7	31.1	43.1	10
Levinko et al.	89.8	85.2	71.8	59.6	71.1	63	53.5	70.6	-
ArtTrack	88.8	87	75.9	64.9	74.2	68.8	60.5	74.3	0.005
Fang et al.	88.4	86.5	78.6	70.4	74.4	73	65.8	76.7	-
Newell et al.	92.1	89.3	78.9	69.8	76.2	71.6	64.7	77.5	-
Fieraru et al.	91.8	89.5	80.4	69.6	77.3	71.7	65.5	78	-
AlphaPose	91.3	90.5	84	76.4	80.3	79.9	72.4	82.1	-
OpenPose	91.2	87.6	77.7	66.8	75.4	68.9	61.7	75.6	0.005

Table S4. The comparison between different pose estimation software on MPII Multi-Person Dataset [S1, S2]

S6. The performance of Mask-Rcnn

In our work, we use the Mask-Rcnn benchmark developed by Facebook to segment people on the images in the videos. In Table S5, it can be seen that Mask-Rcnn performs very well in instance segmentation, although it is slightly inferior to the newer D-SOLO method[S4]. However, the average precision (AP) for person segmentation is very well. Under the Mask-only model, the person's bounding box AP is 53.6, and the person's mask AP is 45.8[S3]. Additionally, the Mask-Rcnn benchmark is based on PyTorch 1.0, which can be compatible with our person classification algorithm. It provides our framework with a lot of convenience for integration and automation. The

pre-training model of people and some common objects has brought us a lot of convenience in our application and supported us in doing a lot of extended research on ASD.

Method	Backbone	mask AP
Mask R-CNN	Res-101-FPN	37.8
TensorMask	Res-101-FPN	37.1
YOLACT	Res-101-FPN	31.2
PolarMask	Res-101-FPN	30.4
SOLO	Res-101-FPN	37.8
D-SOLO	Res-101-FPN	38.4

Table S5. The mask AP for instance segmentation between different methods[S3, S4]. We have compared the popular methods in instance segmentation in recent years and compared their mask AP (average precision, i.e. the Intersection over Union between two masks) under the same backbone on COCO test-dev datasets.

The backbone of the Mask-Rcnn we used in our study is ResNet-152-FPN. Mask-Rcnn is a two-stage method, i.e., detect-and-mask. In our application, we set the threshold for Mask-Rcnn to recognize people to 0.8. This will eliminate some interference, such as the mask image of a hand, a leg, the hair, and so on.

S7. Movement features characteristics at two key points.

We show the characteristics of four MFs of two body key points, e.g., right wrist and middle hip, in five activities in figures. These are shown in Fig. S4 and Fig. S5, including the range and standard deviation of the MFs.

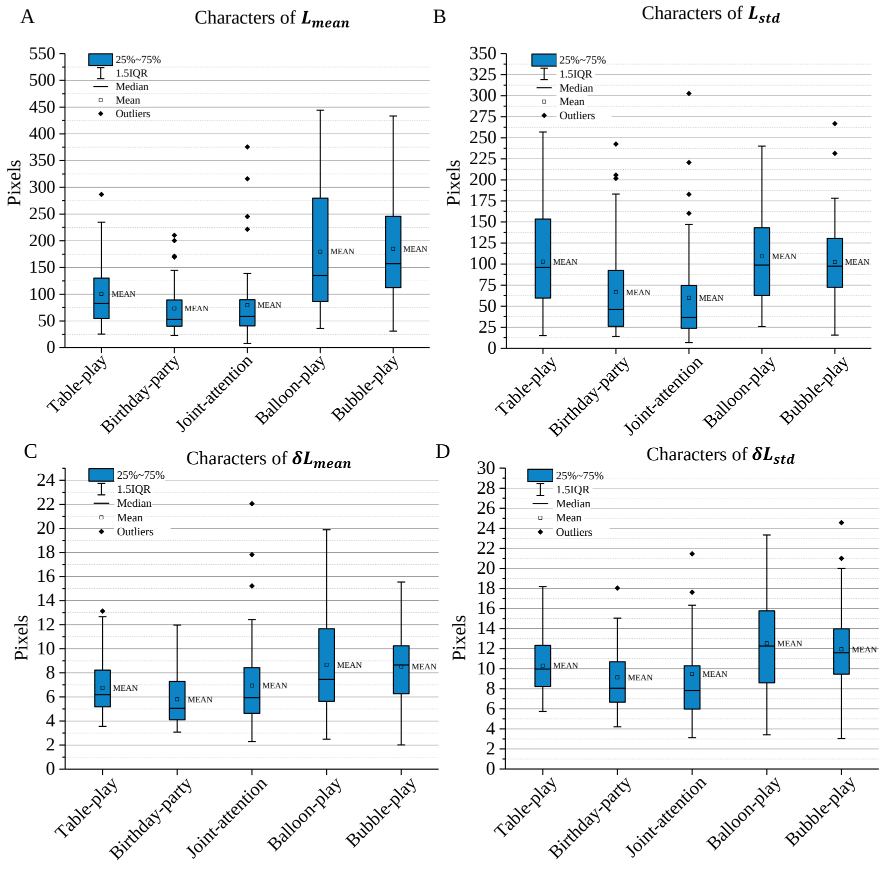

Figure S4. The characteristics of four MFs at the right wrist key point in different activities: (A) L_{mean},

(B) L_{std}, (C) δL_{mean}, and (D) δL_{std}.

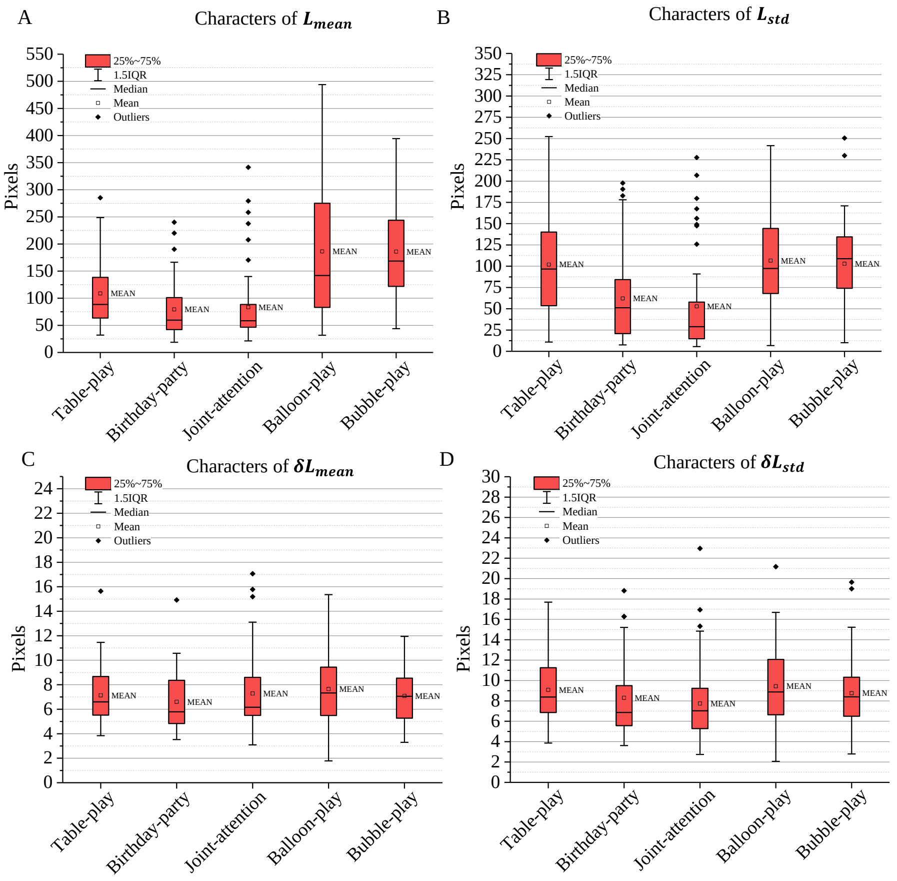

Figure S5. The characteristics of four MFs at the middle hip key point in different activities: (A) L_{mean}, (B) L_{std}, (C) δL_{mean}, and (D) δL_{std}.

S8. Pearson's correlation coefficient (PCC).

We carried out the PCC calculations between different MFs, e.g., L_{mean}, L_{std}, δL_{mean} and δL_{std}, in Table-play for the neck key point, the PCC values are in the range of 0.5 and 0.8, as shown in Table S6. We also carried out the PCC between the same MFs but in different activities for the neck key point, and the results are in the range of 0 to 0.7, see Table S7.

MFs	L_{mean}	L_{std}	δL_{mean}	δL_{std}
L_{mean}	1	0.83	0.84	0.64
L_{std}		1	0.72	0.56
δL_{mean}			1	0.83
δL_{std}				1

Table S6. Pearson's correlation between various MFs of the neck key point in the Table-play activity

MFs	Activities	Table-play	Birthday-party	Joint-attention	Balloon-play	Bubble-play
L_{mean}	Table-play	1	0.58	0.55	0.46	0.28
	Birthday-party		1	0.25	0.49	0.48
	Joint-attention			1	0.04	0.19
	Balloon-play				1	0.45
	Bubble-play					1
L_{std}	Table-play	1	0.55	0.49	0.35	0.44
	Birthday-party		1	0.49	0.38	0.54
	Joint-attention			1	0.09	0.42
	Balloon-play				1	0.56
	Bubble-play					1
δL_{mean}	Table-play	1	0.46	0.57	0.42	0.24
	Birthday-party		1	0.30	0.22	0.38
	Joint-attention			1	0.20	0.06
	Balloon-play				1	0.36
	Bubble-play					1
δL_{std}	Table-play	1	0.76	0.60	0.32	0.18
	Birthday-party		1	0.44	0.23	0.17
	Joint-attention			1	0.22	0.18
	Balloon-play				1	0.33
	Bubble-play					1

Table S7. Pearson's correlation of neck key point in different activities

S9. Correlation power analysis.

We conducted a post hoc power analysis to confirm if we had a sufficient sample size to achieve the study's goal. We used the current sample size as well as the strongest correlations found from the study to calculate power, and set a minimum threshold of $r = 0.50$ for the power analysis. We set the significant level at $p < 0.0125$ (1/4 of a conventional $p < 0.05$ significance level). The results are shown in Table S8.

MFs	Body parts	Sample size	Correlation	Significant level	Power
L_{mean}	neck	52	0.63	0.0125	0.996
L_{std}	neck		0.66		0.998
dL_{mean}	right-wrist		0.67		0.999
Minimum threshold		52	0.50	0.0125	0.914

Table S8. Correlation Power analysis

Reference.

S1. Cao, Z., Simon, T., Wei, S. E., & Sheikh, Y. Realtime multi-person 2d pose estimation using part affinity fields. In *IEEE conference on computer vision and pattern recognition*, 7291-7299 (2017).

S2. Fang, H., et al. Alphapose: Whole-body regional multi-person pose estimation and tracking in real-time. *IEEE Transactions on Pattern Analysis and Machine Intelligence* (2022).

S3. He, K., Gkioxari, G., Dollár, P., & Girshick, R. Mask r-cnn. In *Proceedings of the IEEE*

international conference on computer vision, 2961-2969 (2017).
S4. Wang, X., Kong, T., Shen, C., Jiang, Y., & Li, L. Solo: Segmenting objects by locations. In *European Conference on Computer Vision*, 649-665 (2020).
